# Delayed Saphenous Vein Graft Rupture Secondary to Mediastinal Abscess: A Case Report

**DOI:** 10.7759/cureus.103670

**Published:** 2026-02-15

**Authors:** Oliver M Marigold, Ahmed M Abuaisha, Bradlee Bachar, Hussam Nagm

**Affiliations:** 1 Anesthesiology, Kaweah Health Medical Center, Visalia, USA; 2 Anesthesiology, School of Medicine, University of Tripoli, Tripoli, LBY; 3 Cardiothoracic Anesthesiology, Kaweah Health Medical Center, Visalia, USA

**Keywords:** coronary artery bypass grafting (cabg), graft dehiscence, late graft rupture, mediastinal hemorrhage, saphenous vein graft (svg)

## Abstract

Saphenous vein graft (SVG) rupture is a rare but catastrophic late complication of coronary artery bypass grafting (CABG). We report a 73-year-old male who presented five years after CABG and thoracic aortic aneurysm repair and was found to have a mediastinal fluid collection surrounding the ascending aorta. Emergent surgical exploration under hypothermic circulatory arrest revealed dehiscence of the SVG at its aortic anastomosis with associated mediastinal hemorrhage and evidence of infection at the graft site. The patient required postoperative pharmacologic hemodynamic support and extended antimicrobial therapy for mediastinal infection, followed by gradual clinical recovery. This case highlights the diagnostic challenge and rapid progression of late SVG dehiscence and underscores the importance of early recognition of this rare complication in post-CABG patients presenting with new mediastinal abnormalities.

## Introduction

Saphenous vein graft (SVG) dehiscence and rupture are rare but potentially life-threatening complications in postoperative coronary artery bypass graft (CABG) patients. While early graft failure is common due to thrombosis, hyperplasia, or atherosclerosis, late mechanical complications such as rupture are exceedingly rare [1]. Management is particularly challenging as re-operation comes with a significantly elevated risk of morbidity and mortality [2]. We present a case of a 73-year-old male with a history of CABG who was found to have dehiscence of an SVG off the aorta with subsequent mediastinal hemorrhage five years after his initial operation.

## Case presentation

A 73-year-old male with a past medical history of hypertension, obesity, hyperlipidemia, stroke, and coronary artery disease presented to the emergency department with two weeks of shortness of breath, fatigue, weight loss, fevers, and confusion. He had a cardiac history significant for CABG (left internal mammary artery to left anterior descending artery (LIMA-LAD) and saphenous vein graft to obtuse marginal artery (SVG-OM)) and thoracic aortic aneurysmal repair both in 2019, as well as percutaneous coronary intervention (PCI) with stent placement of the circumflex artery in 2021.

**Figure 1:**
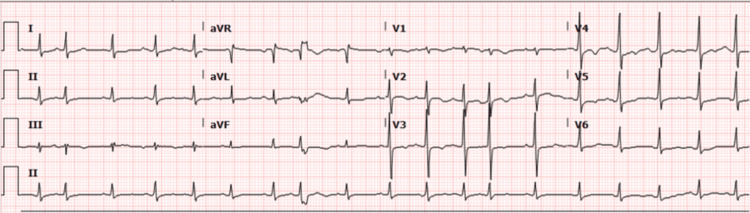
Electrocardiogram showing normal sinus rhythm, premature atrial complexes, and non-specific T wave changes.

**Figure 2:**
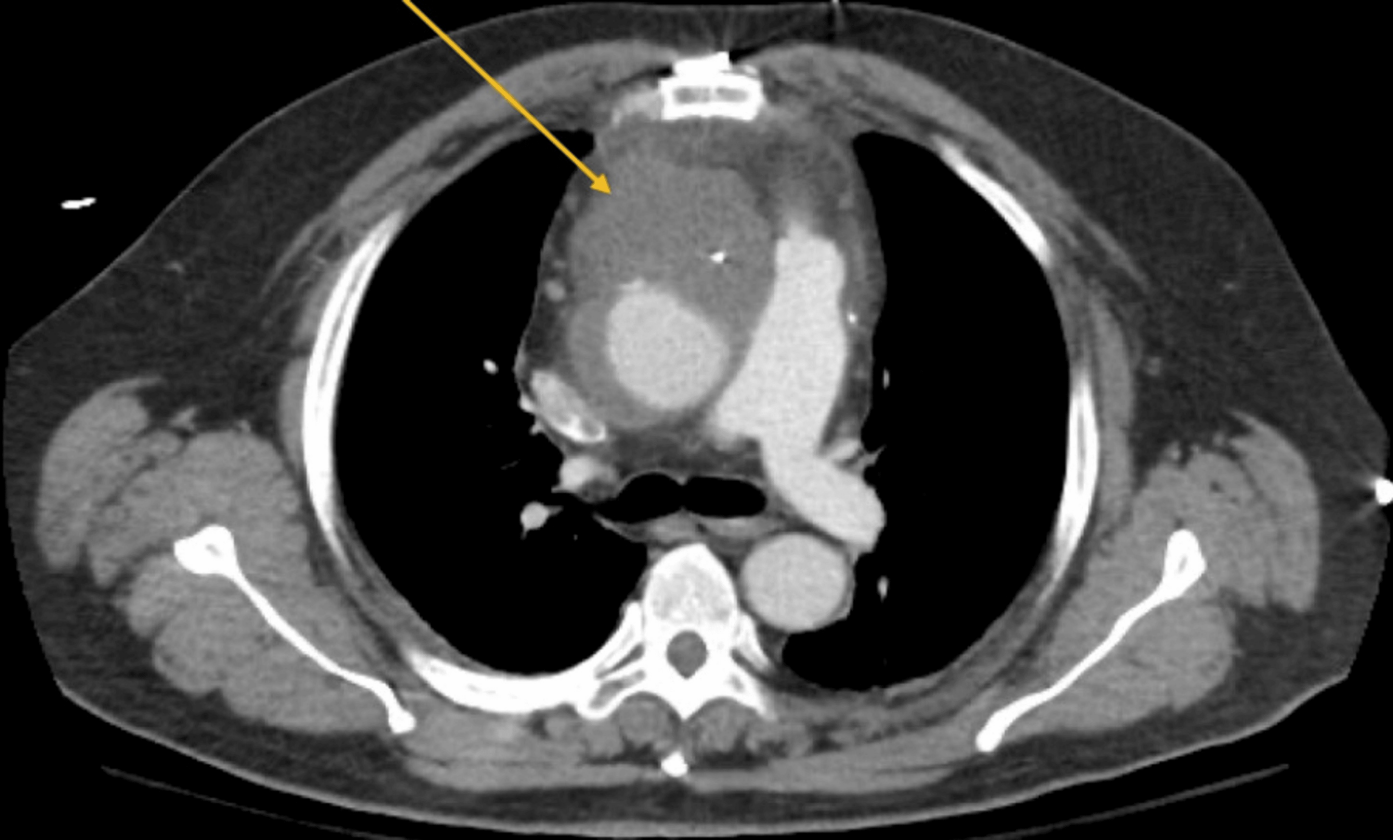
Initial CT imaging of the mediastinal mass.

The patient’s initial vitals showed a temperature of 38.9°C, heart rate of 114 beats per minute, blood pressure of 112/77 mmHg, and oxygen saturation of 95% on 6 liters nasal cannula. Initial workup showed a white blood cell count (WBC) of 20.86 (reference range: 4.00-12.00), high-sensitivity troponin of 4.7 (reference range: 0.000-0.042), and positive respiratory syncytial virus (RSV). The electrocardiogram showed sinus rhythm with premature atrial complexes and non-specific T wave changes (Figure 1). Computed tomography (CT) of the chest with contrast showed hypodense material surrounding the ascending aorta, suspicious for hematoma versus mass (Figure 2). These CT findings did not trigger any acute intervention, and the patient was managed for presumed diagnosis of non-ST-segment elevation myocardial infarction (NSTEMI) secondary to sepsis. He was treated with heparin and antibiotics, and the final blood cultures were positive for group B streptococcus.

**Figure 3:**
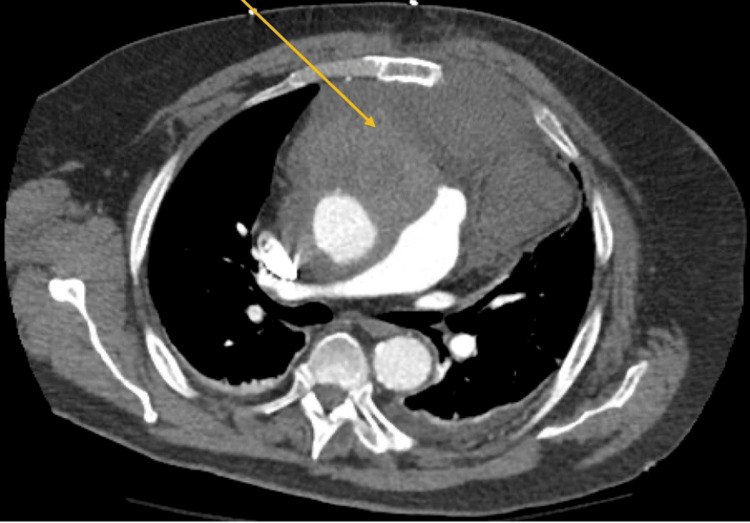
Significant expansion of the mediastinal mass on repeat CT.

On hospital day eight, the patient reported new-onset chest pain and shortness of breath while supine. High-sensitivity troponin was acutely elevated from 0.629 to 5.42, and he was taken for cardiac catheterization, which revealed occlusion of his prior CABG grafts as well as 70-80% mid-LAD stenosis. Given the initial concern for possible hematoma in the setting of a prior aneurysmal repair as well as ongoing shortness of breath, staged PCI of the left anterior descending artery (LAD) was deferred with plans for future intervention once the patient's respiratory symptoms had improved. However, shortly after catheterization, a second chest CT showed significant expansion of the hypodensity surrounding the ascending aorta with an area of active bleeding anteromedially (Figure 3). Cardiac surgery was consulted, and the patient was taken emergently to the operating room.

**Figure 4:**
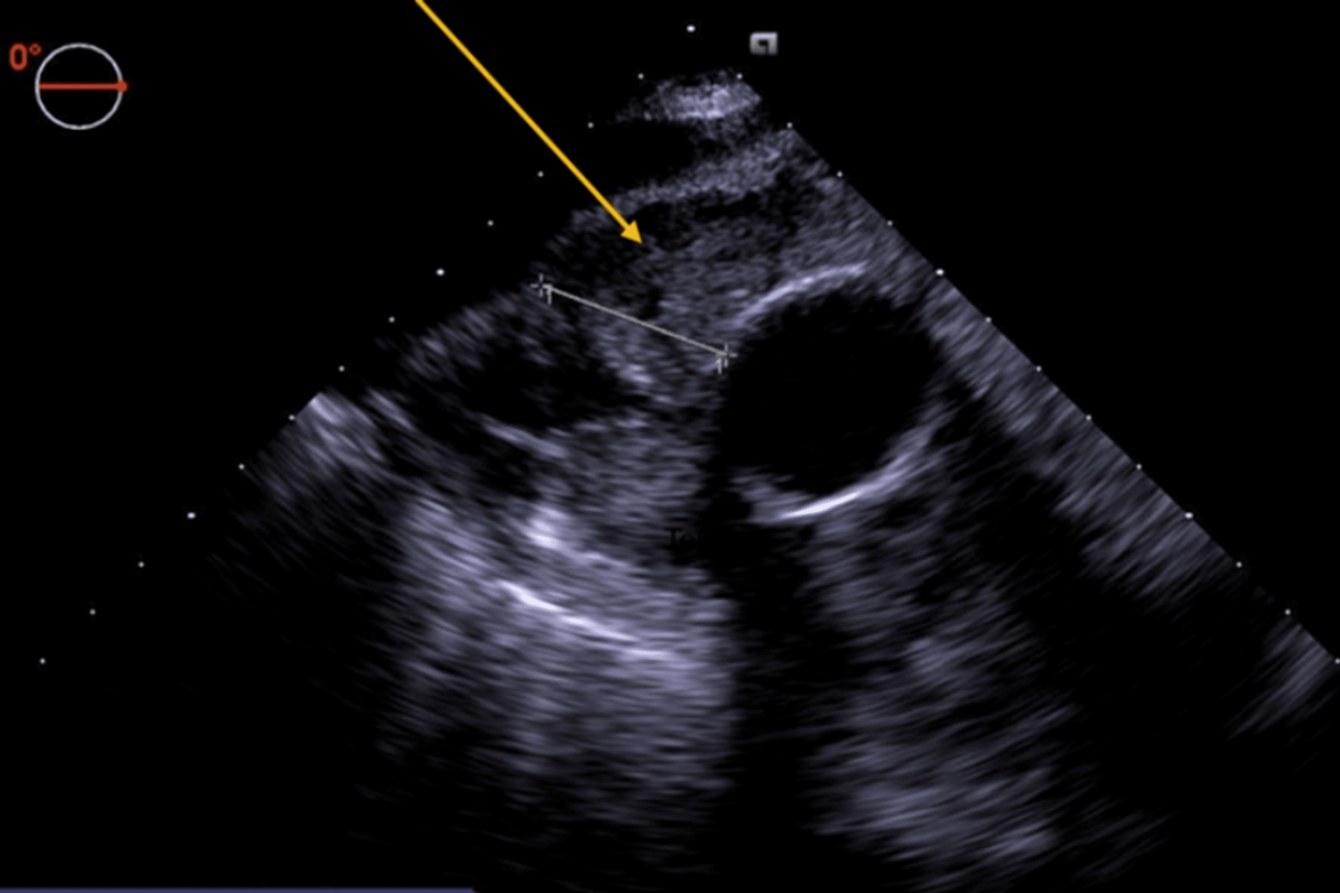
Intraoperative short-axis transesophageal echocardiogram of the mass surrounding the aorta.

**Figure 5:**
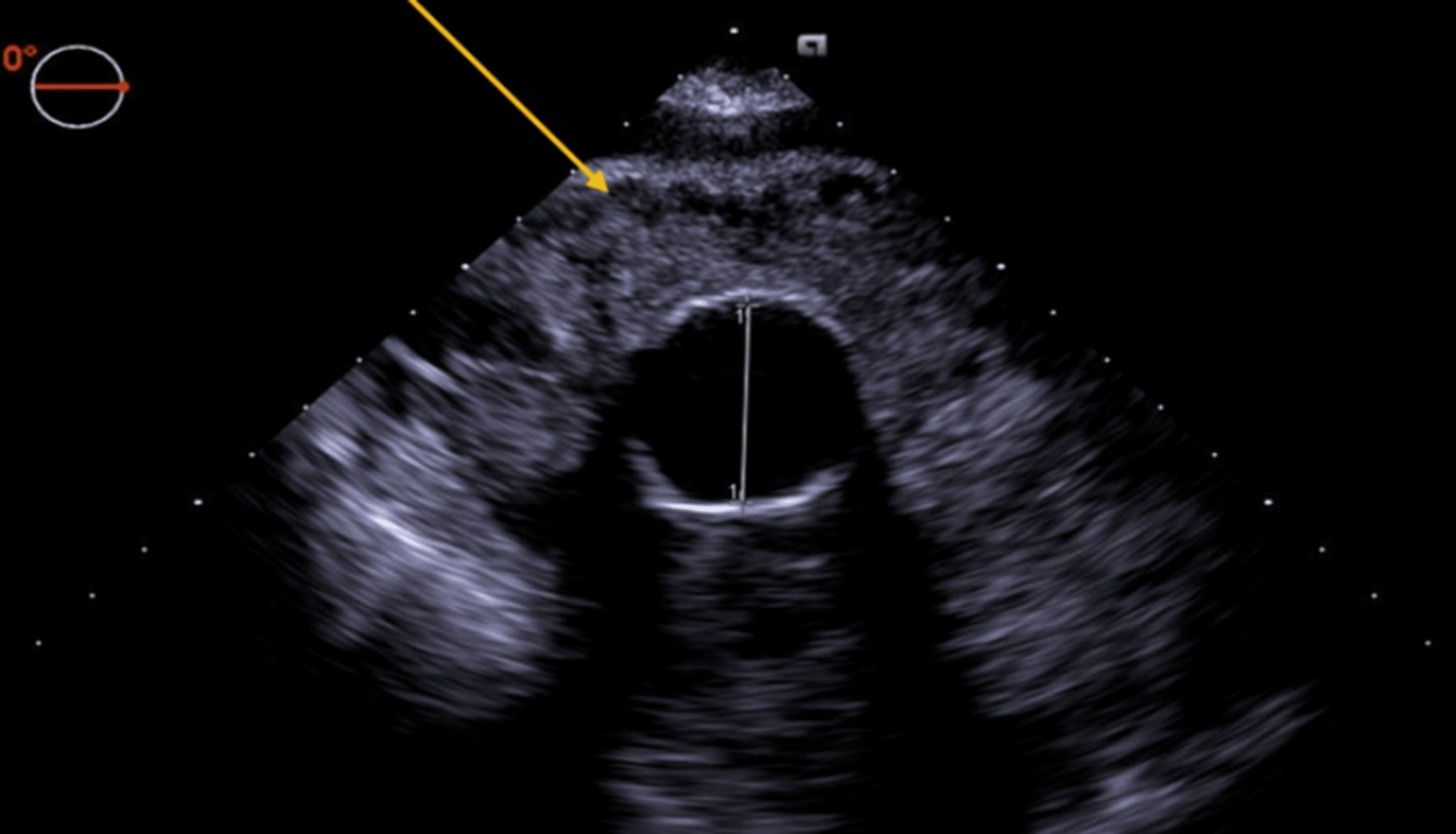
Alternative short-axis transesophageal echocardiogram of the mass.

In the operating room, general anesthesia was induced, and invasive monitoring with a radial arterial line, internal jugular central venous catheter, pulmonary artery catheter, and transesophageal echocardiogram (TEE) was performed. The TEE exam confirmed the presence of a hematoma (Figures 4, 5). Given the unknown etiology of the bleed in the setting of prior thoracic aneurysmal repair, plans were made to perform the procedure under hypothermic circulatory arrest. The patient was femorally cannulated, cooled to 18°C, and placed on cardiopulmonary bypass. Upon surgical exposure, an area of active bleeding was noted at the aortic anastomosis of the venous graft, and an area of purulent material was drained, swabbed, and cultured. Circulatory arrest was initiated and maintained for four minutes.

After adequate rewarming, vasopressors were initiated, and the patient was successfully weaned off bypass. Total cardiopulmonary bypass time was three hours and 50 minutes with a cross-clamp time of approximately 52 minutes. The patient received a total of 1000 mL of recovered autologous blood, two units of packed red blood cells, two units of fresh frozen plasma, and approximately 300 mL of cryoprecipitate. The patient developed right heart failure and vasoplegia in the immediate post-cardiopulmonary bypass period. He was maintained on epinephrine, norepinephrine, and vasopressin infusions as well as inhaled epoprostenol. An intra-aortic balloon pump was placed in the OR and set to a ratio of 1:2.

The patient was transported to the cardiovascular intensive care unit (CVICU) in critical condition with an open chest. His hemodynamic status improved during his CVICU course, and he returned to the operating room on postoperative day two for sternal closure. He was extubated on postoperative day nine. Subsequently, his sternal culture later grew group B streptococcus, matching his initial blood cultures. Repeat chest imaging demonstrated a small abscess anterior to the ascending aorta. The patient was started on an extended course of ceftriaxone. He continued to do well and was eventually downgraded to the acute rehabilitation service and later discharged on lifelong doxycycline treatment. Definitive re-do surgery to address his failed vein grafts was deferred, given the very high operative risk.

## Discussion

SVGs are subject to multiple failure risks in postoperative CABG patients. The primary etiologies are acute thrombosis, intimal hyperplasia, and accelerated atherosclerosis, with up to half of all vein grafts failing by 10 years [1,3]. Rupture of the vein, however, is a rare complication occurring in fewer than 1% of cases. Reported causes include infection, aneurysm, and trauma [4,5]. Infectious cases of graft rupture usually occur in the early postoperative period. Postoperative mediastinitis is a common culprit and has been linked to both vein graft dehiscence and mycotic aneurysm formation [5-7]. Systemic infection has also been implicated, with one report suspecting sepsis from a permanent dialysis catheter as a potential etiology of graft rupture [8]. Instances of late-developing and chronic mediastinal infections, as seen in our patient, are rare. The literature is widely limited to case reports with examples of mediastinitis 15 years after CABG and mediastinal abscess nine years after aortic arch repair [9,10].

Several published reports offer insight into the pathogenesis, management, and outcomes of post-sternotomy infections that may complicate bypass grafts in CABG patients. Diabetes and obesity are thought to be the highest preoperative risk factors, while intraoperative risks include prolonged perfusion time, the use of one or more internal mammary grafts, blood products, and mechanical circulatory support [11]. The overall incidence of mediastinal infections is estimated between 0.3% and 3.4% postoperatively. While the cause of our patient’s infection remains unknown, he did have several identifiable risk factors for mediastinitis, including obesity and the presence of an internal mammary graft. However, due to the rarity of this presenting etiology, vein graft rupture is often not at the top of a differential diagnosis in patients such as ours. Timely diagnosis in these cases is challenging, as seen with our patient, who was initially managed for presumed sepsis with elevated troponin thought to be from type II myocardial infarction. This case highlights the importance of continued monitoring and reevaluation, particularly as symptoms develop or worsen. Prompt repeat troponin, cardiac catheterization, and CT imaging were performed in this case after the patient reported new chest pain and shortness of breath, ultimately leading to the diagnosis and appropriate care. This patient's concurrent coronary artery disease was an additional challenge, as definitive management would have included staged PCI of the LAD. However, given the expansion of his hematoma with active bleeding on CT, he was appropriately triaged for cardiac surgery. This case adds to a small but growing list of similar presentations that serve to aid future diagnostic efforts. As others have stated, vein graft rupture should be considered in a patient with a history of CABG presenting with cardiopulmonary symptoms and a new unidentified mediastinal mass [4].

Given the complexity of the presentation and the medical history in most of these patients, management options depend on the etiology of the graft failure, the acuteness of the illness, hemodynamic stability, and the overall patient presentation. Surgical exploration, antibiotics, PCI, and combined surgical and medical management are all valid options well described in the literature [3]. In our case, the patient was deteriorating rather quickly, and a repeat CT of the chest was highly suspicious for acute dehiscence of the graft from the aorta, pending major bleeding; hence, emergent surgical exploration was mandated. The best way to manage these patients is to prevent initial vein graft failure. Different approaches and regimens are described to optimize and maintain graft patency [1,3]. Preoperatively, a thorough evaluation of the coronary lesion by cardiac angiography and carefully choosing between venous or arterial conduits is essential. Intraoperatively, good harvesting technique and gentle handling of the graft are important to maintain the good quality of the graft. Postoperatively, statins, antiplatelet regimens, close follow-up, early wound debridement, and closure with or without the assistance of vacuum-assisted closure therapy are all important factors in prevention and early management [1,3]. Fortunately, this patient made a good recovery, though will require prolonged antibiotic therapy and ongoing cardiology follow-up.

## Conclusions

Late SVG dehiscence is a rare but potentially life-threatening complication following CABG. This case illustrates the rapid progression and diagnostic challenges associated with mediastinal hemorrhage secondary to graft rupture, particularly in the setting of infection. Early recognition, prompt imaging, and emergent surgical intervention are critical for survival. Clinicians should maintain a high index of suspicion for SVG rupture in post-CABG patients presenting with new mediastinal abnormalities or cardiopulmonary symptoms.
